# *Inonotus obliquus* Polysaccharides Prevent High-Fat Diet-Induced Obesity in Mice Related to Reshaping Gut Microbiota

**DOI:** 10.3390/foods15101769

**Published:** 2026-05-17

**Authors:** Siying Zhang, Ting Liu, Xian Qu, Wenshuo Zhang, Xue Wu, Yadie Liu, Shouchen Li, Dongyuan Shi, Hongyu Li, Aihua Gong

**Affiliations:** 1 Department of Biochemistry, School of Medicine, Jiangsu University, Zhenjiang 212013, China; 2 Luzhou Center for Disease Control and Prevention, Luzhou 646300, China; 3 State Key Laboratory of Cellular Stress Biology, Faculty of Medicine and Life Sciences, School of Life Sciences, Xiamen University, Xiamen 361000, China

**Keywords:** *Inonotus obliquus*, obesity, gut microbiota, functional food ingredient

## Abstract

Obesity development is linked to disturbances in the gut microbiota. *Inonotus obliquus* polysaccharides (IOPs) have potential therapeutic efficacy in alleviating metabolic disorders. However, the mechanism by which IOP prevents obesity via regulating gut microbiota remains elusive. IOP was extracted and structurally characterized by FT-IR and NMR spectroscopy, confirming typical polysaccharide structures. Structurally, IOP is a 5.4 kDa polysaccharide predominantly composed of glucose, galactose, xylose, mannose, galacturonic acid, glucuronic acid, as well as rhamnose, arabinose, and methyl-galactose. Administration of IOP to high-fat diet (HFD)-fed mice effectively curtailed weight gain and improved serum lipid parameters. Furthermore, it mitigated lipid deposition within hepatic and adipose tissues, while successfully countering HFD-triggered liver damage. Notably, IOP induced significant changes in microbial diversity and composition by selectively increasing the abundance of *Streptococcaceae* while suppressing *Faecalibaculum rodentium* at the family and species levels. These findings highlight that IOP is a promising functional food ingredient that regulates gut microbiota for obesity prevention.

## 1. Introduction

Obesity is a pervasive metabolic disorder characterized by a sustained surplus of energy intake relative to expenditure [1]. Beyond its status as a global health challenge affecting all age groups, it acts as a primary driver for numerous chronic conditions, ranging from cardiovascular diseases and type 2 diabetes to osteoarthritis and various cancers [2]. Childhood and adolescent obesity have become an intensifying worldwide public health threat, and it is predicted that more than 250 million young individuals will be affected by 2030 [3]. Particularly, studies have confirmed that obese children and adolescents are prone to persistent obesity in adulthood without timely intervention [4]. These epidemiological data highlight the urgency of exploring effective strategies for preventing and managing obesity.

Accumulating evidence indicates that obesity is tightly linked to gut microbiota dysbiosis [5,6]. Considered a crucial “metabolic organ” of the host, the gut microbiota plays a pivotal role in the assimilation of nutrients, preservation of caloric equilibrium, and immune response modulation [7]. Gut dysbiosis is primarily characterized by alterations in microbial diversity, aberrant abundance of specific taxa, and metabolic dysfunction. This state is often associated with a reduced synthesis of health-promoting metabolites, notably short-chain fatty acids (SCFAs), and may promote low-grade chronic inflammation, thereby potentially exacerbating obesity and related metabolic disorders [8]. Consequently, therapeutic interventions focusing on gut microbial regulation have surfaced as a potential avenue for obesity intervention. Although synthetic drugs are currently available for metabolic regulation, extended administration is frequently correlated with deleterious complications, such as gastrointestinal discomfort and drug resistance, resulting in poor patient compliance [9]. These limitations highlight the urgent need for safe and effective natural interventions. Among these, polysaccharides from medicinal fungi have been shown to improve host metabolic disorders by regulating gut microbiota homeostasis, representing a class of natural products with substantial development potential [10,11].

*Inonotus obliquus* (Chaga) has been utilized in the ethnomedicine of Russia, China, and Korea for centuries, where it is traditionally employed to manage cardiovascular and metabolic diseases [12]. Among its diverse bioactive components, *Inonotus obliquus* polysaccharide (IOP) has been identified as a core bioactive agent. IOP possesses diverse medicinal functions, including anti-tumor [13], anti-inflammatory [14], and antioxidant effects [15], as well as significant hypoglycemic [16] and hypolipidemic activities [17], all while maintaining a favorable safety profile [18]. Growing evidence suggests the therapeutic potential of IOP in obesity intervention. For instance, Li Yang et al. demonstrated that after intragastric administration, IOP could improve lipid metabolism disorders in hyperlipidemic mouse models by facilitating the generation of SCFAs and modulating the composition of intestinal microbiota [17]. Yiming Guo et al. found that after intragastric administration, IOP could improve insulin insensitivity within skeletal muscle in HFD/STZ-induced type 2 diabetes mellitus (T2DM) mouse models, and this mechanism of action might be associated with the PI3K/AKT and AMPK/ACC1/CPT1 pathways [19]. Collectively, these findings support the potential efficacy of IOP in treating and controlling obese conditions.

However, current research has predominantly focused on the therapeutic effects of IOP on established obesity, largely overlooking its preventive potential regarding the onset of the disease. Furthermore, existing data are derived primarily from experimental administration via intragastric gavage, which limits the translational potential of IOP as a functional food ingredient. Additionally, the specific mechanisms by which IOP modulates the gut microbiota during the development of obesity remain to be fully elucidated. To bridge these gaps in knowledge, the present study targets gut microbiota modulation to evaluate the preventive role of IOP against obesity. Specifically, IOP was administered via an HFD to explore its potential as a functional food ingredient, while the associated mechanisms were systematically analyzed on gut microbiota. The originality of this study lies in its preventive design, the dietary administration of IOP mixed with an HFD, and the focused investigation on gut microbiota modulation. The findings of this study are expected to provide new insights into the mechanisms underlying the anti-obesity effects of IOP and offer solid data to support the development and transformation of natural bioactive ingredients into functional food products.

## 2. Materials and Methods

### 2.1. Materials and Reagents

*Inonotus obliquus* was purchased from Bozhou Xiangxin Pharmaceutical Co., Ltd. (Bozhou, China).Dextran standards and standard monosaccharides were all procured from Sigma (St. Louis, MO, USA). High-fat diet was supplied by Changzhou Shuyishuer Biotechnology Co., Ltd. (Changzhou, China). All commercial assay kits were purchased from Nanjing Jiancheng Bioengineering Institute (Nanjing, China). Additional chemicals employed were of analytical purity and originated from Beijing Chemical Works (Beijing, China).

### 2.2. Preparation of IOP

IOP was prepared as follows: The fruit bodies were fragmented into minor portions and immersed in 95% ethanol overnight to remove alcohol-soluble small-molecule impurities. Subsequently, the treated samples were blended with deionized water, maintaining a 1:30 ratio of solids to liquids, and then extracted with boiling water three times (2 h each time). The filtrate was centrifuged at 4500 rpm/min for 15 min. After performing concentration under vacuum at 60 °C, the supernatant was treated with a triple volume of 95% ethanol. The solution was then kept at 4 °C overnight to allow for the precipitation of crude polysaccharides. The sample was further subjected to hollow fiber separation (3 kDa) to remove small-molecule impurities. The retained macromolecular fraction was collected and the target IOP was obtained through a freeze-drying process.

### 2.3. Structural Characterization of IOP

#### 2.3.1. Analysis of Total Sugars and Uronic Acids

The concentration of total polysaccharides was evaluated via an adapted phenol-sulfuric acid colorimetric approach, employing glucose as the reference standard and absorbance was measured at 490 nm [20,21]. To quantify the glycuronic acid content, a modified m-hydroxybiphenyl technique was utilized, with galacturonic acid serving as the standard, employing galacturonic acid as the standard, and absorbance was recorded at 520 nm [22]. All absorbance measurements were performed using visible spectrophotometer (Shanghai Spectrum Instrument Co., Ltd., Shanghai, China, Model 721E).

#### 2.3.2. Measurement of Molecular Weight

High-performance gel permeation chromatography (HPGPC) was performed to determine the molecular weight distribution. The instrumentation consisted of a Shimadzu HPLC system (Shimadzu, Kyoto, Japan) equipped with an LC-20Ai delivery pump, a RID-20A differential refractive index detector, and a TSK-gel G3000PWXL column (7.8 × 300 mm). Before being loaded into the instrument, the sample was diluted to 5 mg/mL, and then subjected to impurity removal via filtration with a 0.22 μm aqueous-phase membrane filter. The optimized chromatographic parameters for the assay were set as follows: 0.2 M NaCl solution was used as the mobile phase, with a constant elution flow rate set to 0.6 mL/min; the temperature of both the column oven and the detector was maintained at 40 °C throughout the test, and the injection volume for each sample was fixed at 20 μL.

#### 2.3.3. Monosaccharide Composition Analysis

Monosaccharide composition of IOP was characterized via HPLC as previously described [23]. Briefly, 1 mg of IOP was subjected to hydrolysis using 2 M methanolic hydrochloride solution at 80 °C for 16 h, and then underwent a secondary hydrolysis with trifluoroacetic acid (TFA) at 120 °C for 1 h. The monosaccharides released from the hydrolysis were derivatized with 1-phenyl-3-methyl-5-pyrazolone (PMP) as the derivatization reagent. The chromatographic analysis was carried out on a Shimadzu HPLC system (Shimadzu, Kyoto, Japan), which was configured with an LC-20AT delivery pump, an SPD-20A ultraviolet (UV) detector, and a COSMOSIL 5C18-PAQ analytical column (4.6 × 250 mm). All test samples were filtered through a 0.22 μm membrane filter before chromatographic injection. The optimized chromatographic parameters for the assay were set as follows: the mobile phase was composed of 81.5% 0.1 M phosphate-buffered saline (PBS, pH 7.0) and 18.5% acetonitrile (*v*/*v*); the flow rate was set at 1.0 mL/min; the column temperature was controlled at 35 °C; the detection wavelength was fixed at 245 nm; and the injection volume for each sample was 10 μL.

#### 2.3.4. Fourier-Transformed Infrared (FT-IR) Analysis

To capture the FT-IR absorption profiles of IOP, a Spectrum Two instrument (PerkinElmer, Waltham, MA, USA) was employed. Briefly, 1 mg of the specimen was homogenized with desiccated KBr powder and pulverized into a fine consistency before being compacted into 1 mm pellets. Measurements were conducted across an operational window encompassing wavenumbers from 4000 to 400 cm^−1^.

#### 2.3.5. Nuclear Magnetic Resonance (NMR) Analysis

To perform NMR analysis, 20 mg of IOP was dissolved in 0.5 mL of 99.8% D_2_O. Both ^1^H NMR and ^13^C NMR spectra were recorded at 25 °C using a Bruker Avance 600 MHz spectrometer (Bruker, Karlsruhe, Germany) [24]. Data processing was executed via standard Bruker software. Chemical shifts were expressed in ppm using acetone chemical shift at ^1^H/^13^C 2.17/29.20 ppm as an internal standard.

### 2.4. Animal Experimental Design

All animal-related procedures were strictly aligned with the National Institute of Health Guide for the Care and Use of Laboratory Animals and were authorized by the Jiangsu University Ethics Committee (Approval No.: UJS-IACUC-AP-2023030332). Six-week-old male C57BL/6J mice were purchased from Beijing Vital River Laboratory Animal Technology Co., Ltd. and maintained at the Jiangsu University animal center. The rearing environment was stabilized at 22.0 ± 0.5 °C with 55 ± 5% humidity and a 12 h light/dark cycle, providing the mice with ad libitum access to food and water. Following a 7-day acclimatization period, the mice were randomly allocated into four cohorts (n = 6 each) for a 13-week experimental duration. The Control group was fed a standard diet (12% kcal from fat, 24% kcal from protein, 64% kcal from carbohydrates), while the HFD group received a high-fat diet (60% kcal from fat, 20% kcal from protein, 20% kcal from carbohydrates). The remaining groups were administered the high-fat diets supplemented with either 0.1% (*w*/*w*) or 0.2% (*w*/*w*) IOP. Energy intake (kcal/day/mouse) was calculated by multiplying the mass of food consumed (g) by the energy density of the respective diet: 5.24 kcal/g for the HFD and 3.85 kcal/g for the Control diet. Body weight measurements were taken weekly. Before sacrifice, all animals were subjected to an overnight fast. Blood was harvested through orbital extraction, followed by euthanasia via cervical dislocation. For subsequent analysis, liver tissues and gonadal white adipose tissue (gWAT) were collected and fixed in 4% paraformaldehyde. Colonic contents were immediately flash-frozen in liquid nitrogen and kept at −80 °C.

### 2.5. Hematoxylin–Eosin (HE) Staining

For histological analysis, gWAT and liver samples were initially fixed in 4% paraformaldehyde for 24 h. Subsequently, the tissues were dehydrated, embedded in paraffin, and sectioned at a thickness of 5 μm for Hematoxylin and Eosin (H&E) staining. A Nikon light microscope was employed to observe the morphological characteristics of the tissues.

### 2.6. Analysis of Serum and Hepatic Biochemical Indicators

Blood samples from mice were kept at 4 °C for 20 min and then centrifuged at 1000× *g* for 15 min to obtain serum. Liver tissues were rinsed with normal saline, homogenized in 0.9% saline, and the supernatant was collected for further assays. Commercial kits from Nanjing Jiancheng Bioengineering Institute (Nanjing, China) were used to measure serum triglycerides (TG), total cholesterol (TC), non-esterified fatty acids (NEFAs), alanine transaminase (ALT) and aspartate transaminase (AST).

### 2.7. 16S rRNA Gene Sequence and Analysis

Fecal microbial DNA was extracted from colonic contents using the CTAB method. DNA integrity and concentration were verified by 1% agarose gel electrophoresis. Amplification of the 16S rRNA V3–V4 region was performed, followed by sequencing on an Illumina NovaSeq platform (250 bp paired-end reads) at Shanghai Personal Biotechnology Co., Ltd. (Shanghai, China). Raw reads were demultiplexed, merged using FLASH (v1.2.7), and quality-filtered. Chimeric sequences were identified and removed via the UCHIME algorithm against the Silva database. Effective tags were clustered into operational taxonomic units (OTUs) using Uparse (v7.0.1001) at 97% similarity. OTU abundance was normalized to the sample with the smallest sequence count. Alpha- and beta-diversity were computed using QIIME (v1.7.0 and v1.9.1, respectively), with data visualization performed in R (v4.1.1) and GraphPad Prism 10.0. Analyses were also supported by the GenesCloud platform (https://www.genescloud.cn/home; accessed on 29 March 2026).

### 2.8. Statistical Analysis

Statistical analyses were conducted using GraphPad Prism 10.0 (GraphPad Software, San Diego, CA, USA). All data are presented as mean ± s.e.m. The normality and homogeneity of variances were verified for all data meeting the assumptions of parametric tests. All analyses were based on the same number of biological replicates (n = 6). Comparisons among multiple groups were performed using one-way analysis of variance (ANOVA), followed by Dunnett’s multiple comparisons test as the post hoc test. A *p*-value of less than 0.05 was considered statistically significant. The associations between gut microbial taxa and host metabolic parameters were evaluated using Spearman’s correlation analyses. *p*-values are corrected by the Benjamini–Hochberg (FDR) approach. The level of significance is indicated by stars in each square.

## 3. Results

### 3.1. Physicochemical Composition Analysis

#### 3.1.1. The Polysaccharide Contents

The extraction yield of IOP was 8.1%. Its total carbohydrate content, quantified by the phenol-sulfuric acid colorimetric method, reached 46.3%, while the uronic acid content was determined to be 18.6% using the m-hydroxybiphenyl assay.

#### 3.1.2. Molecular Weight (Mw) and Monosaccharide Composition Analysis

**Figure 1:**
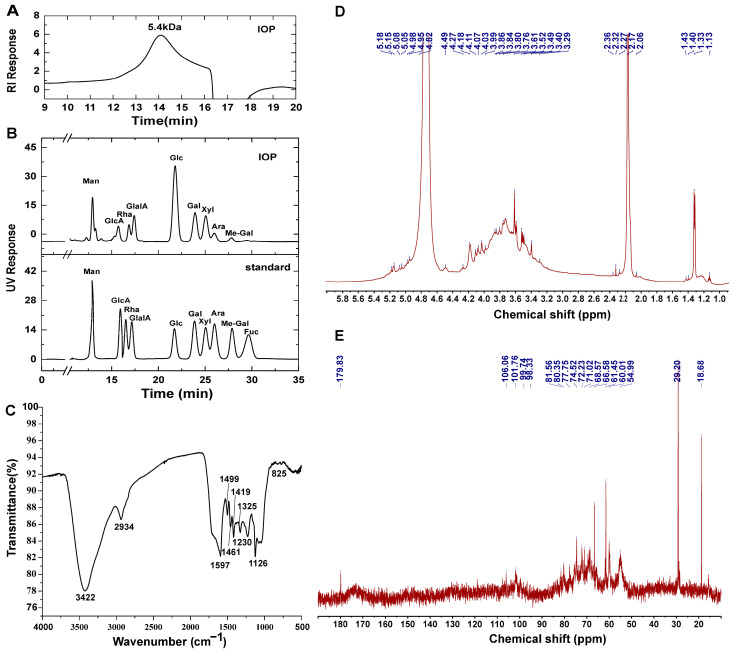
The structural characteristics of IOP. Molecular weight distribution of IOP determined by HPGPC (**A**). Monosaccharide composition of IOP determined by HPLC (**B**). FT-IR spectrum of IOP (**C**). ^1^H NMR spectrum of IOP (**D**). ^13^C NMR spectrum of IOP (**E**). Abbreviations: Man (mannose), GlcA (glucuronic acid), Rha (rhamnose), GalA (galacturonic acid), Glc (glucose), Gal (galactose), Xyl (xylose), Ara (arabinose), Me-Gal (methyl-galactose).

As presented in Figure 1A, the molecular weight and homogeneity of IOP were assessed by HPGPC, which revealed a single dominant peak at 5.4 kDa. Subsequent monosaccharide composition analysis via HPLC (Figure 1B) demonstrated that IOP comprised nine monosaccharides, with glucose (Glc, 37.2%) as the predominant component, followed by galactose (Gal, 13.9%), xylose (Xyl, 12.6%), mannose (Man, 10.0%), galacturonic acid (GalA, 9.8%), glucuronic acid (GlcA, 5.7%), rhamnose (Rha, 4.9%), arabinose (Ara, 4.3%), and a minor amount of methyl-galactose (Me-Gal, 1.6%).

#### 3.1.3. FT-IR Analysis

As presented in Figure 1C, the FT-IR spectrum of IOP revealed several key absorption bands characteristic of polysaccharides. The intense broad band at 3422 cm^−1^ was assigned to O–H stretching vibrations, indicating abundant hydroxyl groups. The absorption at 2934 cm^−1^ corresponded to C–H stretching, suggesting the presence of methyl and methylene groups. A prominent band at 1597 cm^−1^ was attributed to C=O stretching, confirming the carboxyl groups and supporting the acidic nature of IOP. Additional bands at 1499, 1461, and 1419 cm^−1^ were associated with C–H bending of methylene groups (–CH_2_–), while peaks at 1325 and 1230 cm arose from C–OH deformation vibrations. The signal at 1126 cm^−1^ confirmed the presence of C–O–C glycosidic linkages, characteristic of polysaccharide backbones. Furthermore, the absorption at 825 cm^−1^ indicated α-anomeric configurations [25]. Collectively, these spectral features are in agreement with those reported for bioactive polysaccharides and provide structural confirmation of IOP [26].

#### 3.1.4. NMR Analysis

In the ^1^H NMR spectrum, distinct anomeric proton signals were observed in the region of δ 4.4–5.2 ppm. Signals at δ 5.18–4.95 ppm were assigned to α-configured anomeric protons, while resonances at 4.82 and 4.49 ppm were attributed to β-configured anomeric protons [27]. In the ^13^C NMR spectrum, anomeric carbon signals were detected in the range of δ 90–110 ppm, further confirming the presence of multiple glycosidic linkages. The signal at 179.83 ppm was attributed to carboxyl carbon atoms, indicating the presence of uronic acid units and supporting the acidic nature of IOP [28]. Signals at δ 106.06 and 101.76 ppm were assigned to β-anomeric carbons, whereas those at δ 99.74 and 98.33 ppm corresponded to α-anomeric carbons. The signal at 54.99 ppm was attributed to carbon signal of O-CH_3_ group [29], which is in good agreement with the presence of Me-Gal in IOP. These findings suggested that IOP contained both α- and β-pyranose configurations, which were consistent with the structural features of related polysaccharides reported in the literature [30].

### 3.2. IOP Intervention Attenuates Weight Gain and Fat Accumulation in HFD-Fed Mice

**Figure 2:**
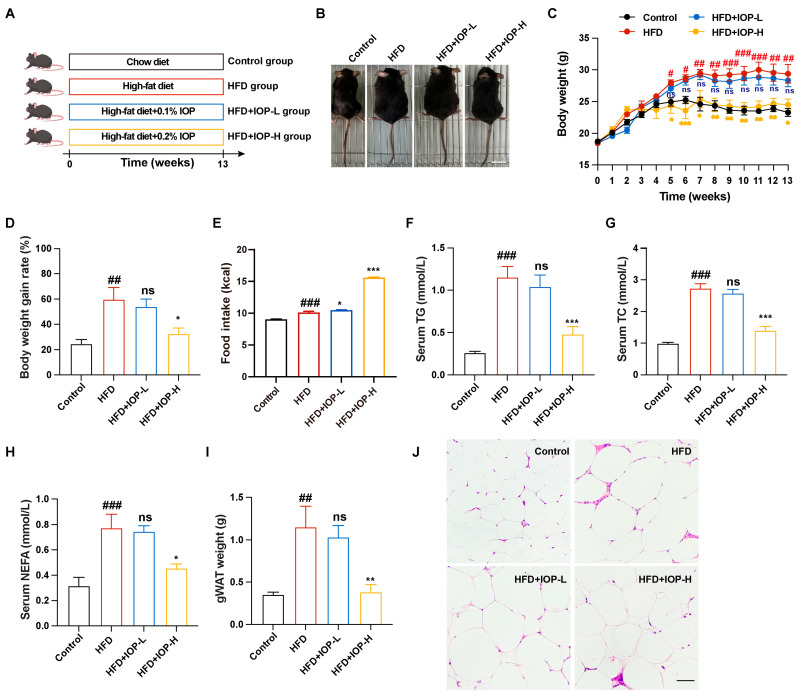
Effects of IOP intervention on body weight and fat accumulation in HFD-fed mice. Experimental design, n = 6 (**A**). Images of mice appearance, Scale bar: 2 cm (**B**). Body weight (**C**). Body weight gain rate (**D**). Food intake (**E**). The content of TG (**F**), TC (**G**) and NEFA (**H**) in serum. gWAT weight (**I**). H&E staining images of gWAT, Scale bar: 100 μm (**J**). # *p* < 0.05, ## *p* < 0.01, ### *p* < 0.001 compared with the Control group. * *p* < 0.05, ** *p* < 0.01, *** *p* < 0.001 compared with the HFD group. ns, not significant.

To investigate the preventive effect against obesity of IOP, C57BL/6 mice were distributed into four distinct groups through random allocation and underwent a 13-week feeding regimen (Figure 2A): the Control group (fed a standard chow diet), the HFD group (fed a high-fat diet), and two IOP-intervention groups (fed a HFD supplemented with IOP at doses of 0.1% or 0.2%, respectively). As shown in Figure 2B–D, compared with the Control group, mice in the HFD group exhibited a significant increase in body weight. In contrast, supplementation with high (0.2%, IOP-H) doses of IOP effectively ameliorated the HFD-induced obese phenotype, resulting in a notable decline in both body weight and body weight gain rate of the obese mice (Figure 2C,D). Notably, food intake was significantly higher in the IOP-H group than in the HFD group (Figure 2E), suggesting that the weight-lowering effect of IOP was unlikely to be explained by reduced food intake alone. Further analysis of serum lipid profiles demonstrated that HFD administration triggered a substantial rise in serum concentrations of TG, TC, and NEFA in comparison to the Control group (Figure 2F–H). However, high doses of IOP significantly decreased the serum levels of these lipids (Figure 2F–H). Furthermore, high doses of IOP significantly alleviated lipid accumulation in gWAT and reduced adipocyte size in HFD-fed mice (Figure 2I,J). In contrast, low-dose IOP (0.1%, IOP-L) showed no significant effect on improving HFD-induced obesity. Collectively, these results demonstrated that IOP exerted a significant anti-obesity effect, which was dependent on its dosage.

### 3.3. IOP Ameliorates Hepatic Steatosis and Hepatic Injury

**Figure 3:**
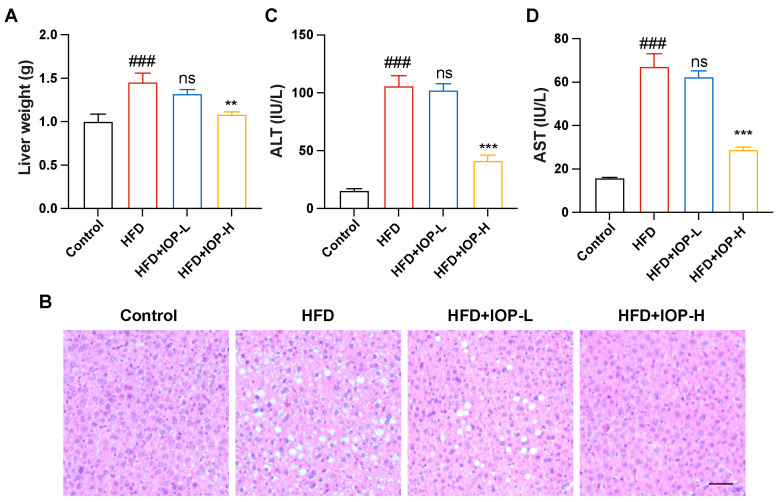
Effects of IOP intervention on hepatic steatosis and liver injury. Liver weight (**A**). H&E staining images of liver tissue, scale bar: 100 μm (**B**). The serum levels of ALT (**C**) and AST (**D**). ### *p* < 0.001 compared with the Control group. ** *p* < 0.01, *** *p* < 0.001 compared with the HFD group. ns, not significant.

Lipid metabolic disturbances provoked by HFD are recognized as critical contributors to hepatic steatosis [31]. In this study, HFD consumption resulted in increased liver weight and pronounced lipid droplet deposition in the liver, both of which were ameliorated by high doses of IOP intervention (Figure 3A,B). To assess liver function, two key biomarkers of hepatic injury were measured. Notably, HFD feeding markedly elevated the levels of ALT and AST in serum, indicating impaired liver function. In contrast, high doses of IOP interventions abrogated these HFD-induced elevations (Figure 3C,D). Mirroring the findings in adipose tissue, low-dose IOP (IOP-L) did not confer significant protection against HFD-induced hepatic abnormalities (Figure 3A–D). Taken together, these results demonstrated that IOP attenuated liver abnormalities associated with high-fat diet feeding.

### 3.4. IOP Changes the Structure of Gut Microbiota

**Figure 4:**
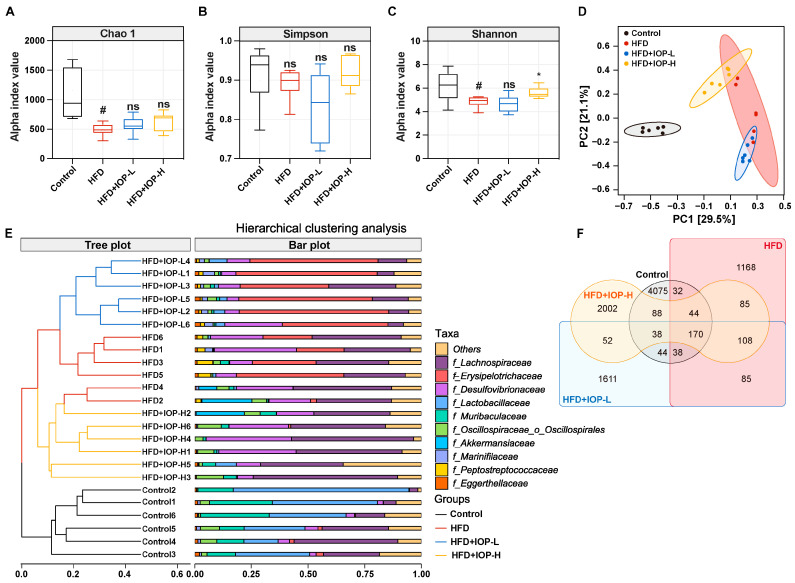
Effects of IOP treatment on gut microbiota diversity in HFD-induced obese mice. Alpha diversity was evaluated using Chao1 (**A**), Simpson (**B**) and Shannon (**C**). Beta-diversity was visualized via PCoA (**D**) and UPGMA (**E**) based on unweighted UniFrac distances. Venn diagram illustrating the number of unique and shared operational taxonomic units (OTUs) among groups (**F**). Data are presented as mean ± SEM (n = 6). # *p* < 0.05 compared with the Control group. * *p* < 0.05 compared with the HFD group. ns, not significant.

Given the established link between obesity and gut microbiota dysbiosis, and considering that orally administered polysaccharides often exert their effects by modulating the gut microbiota rather than through direct host interaction [32]. Hence, we performed 16S rRNA sequencing to assess the impact of IOP on the gut microbiota. Consistent with previous reports, HFD markedly depressed the α-diversity of the gut microbiota compared with the Control group (Figure 4A–C). Oral administration of the high-dose IOP (HFD+IOP-H), but not the low-dose (HFD+IOP-L), significantly elevated Simpson’s diversity index (*p* < 0.05), whereas neither Chao1 nor Shannon indices were altered (Figure 4A–C). These data indicate that IOP specifically mitigates the HFD-induced loss of microbial diversity and evenness without affecting richness.

β-diversity was evaluated using PCoA and UPGMA clustering based on unweighted UniFrac distances. A distinct separation of microbial communities was observed between the Control and HFD groups (Figure 4D,E). Importantly, high-dose IOP treatment shifted the gut microbiota composition of HFD-fed mice toward that of the Control group (Figure 4D,E). Venn diagram analysis revealed 3662 shared OTUs across all groups, with unique OTU counts of 4075 (Control), 1168 (HFD), 1161 (HFD+IOP-L), and 2002 (HFD+IOP-H) (Figure 4F). Collectively, these results demonstrate that high-dose IOP reshapes the gut microbiota structure in HFD-fed mice.

### 3.5. IOP Changes the Composition of Gut Microbiota

**Figure 5:**
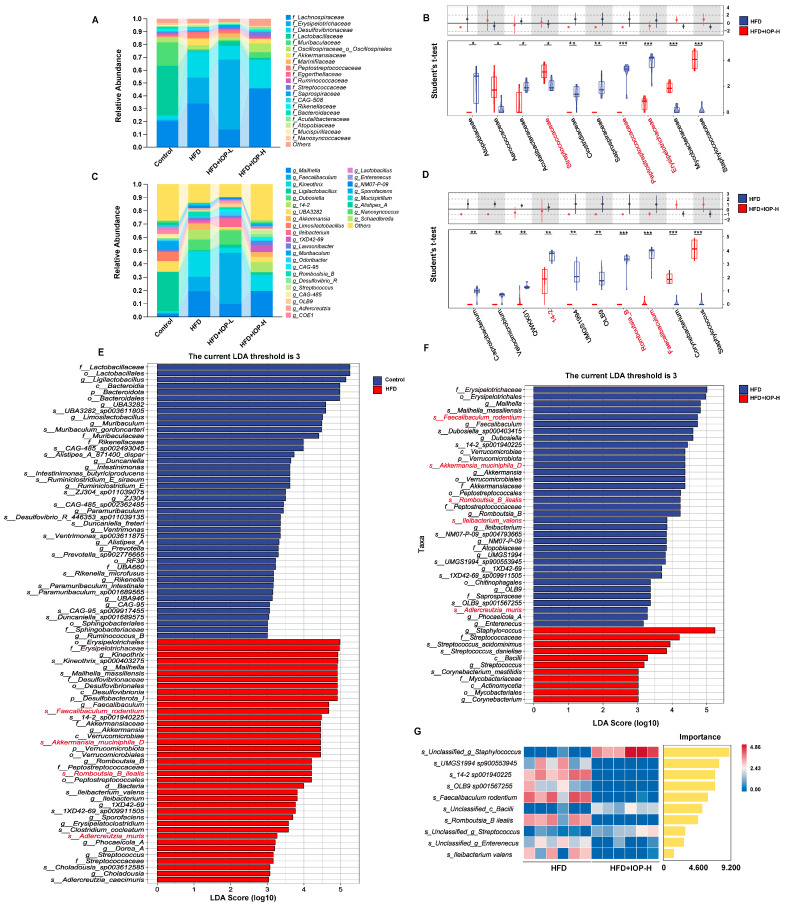
Effect of IOP treatment on gut microbiota composition in HFD-induced obese mice. Relative abundance of the top 20 ranked bacterial families in each group (**A**). Student’s *t*-test bar plot in the gut microbiota of the HFD group and HFD+IOP-H group at the family (**B**). Relative abundance of the top 30 ranked bacterial genera in each group (**C**). Student’s *t*-test bar plot in the gut microbiota of the HFD group and HFD+IOP-H group at the genus level (**D**). Linear discriminant analysis (LDA) coupled with effect size (LEfSe) analysis revealing significantly differentially abundant operational taxonomic units (OTUs) between the HFD group and the Control group. Only taxa exceeding an LDA score threshold of 3.0 with statistical significance (*p* < 0.05, Kruskal–Wallis test followed by pairwise Wilcoxon rank-sum test) are presented (**E**). LEfSe analysis identifying differentially abundant OTUs in the HFD+IOP-H group relative to the HFD group at the OTU level (**F**). Top 10 biomarkers discriminating the HFD group from the HFD+IOP-H group as identified by random forest analysis at the species level. Species are ranked according to their mean decrease in accuracy (MDA) values (**G**). * *p* < 0.05, ** *p* < 0.01, *** *p* < 0.001 compared with the HFD group.

All the above results demonstrate that IOP markedly reshaped the structure of the gut microbiota. To elucidate the underlying taxonomic shifts, we decomposed the community structure at the family, genus and species levels. Stacked-bar profiling revealed that, relative to the Control group, HFD profoundly perturbed both family- and genus-level community composition (Figure 5A,C), indicative of dysbiosis. Strikingly, dysbiosis was partially reversed only in mice receiving the high-dose IOP (IOP-H) but not IOP-L. IOP-H selectively elevated the abundance of *Streptococcaceae* (*p* < 0.05) while concomitantly suppressing *Saprospiraceae* (*p* < 0.01), *Peptostreptococcaceae* and *Erysipelotrichaceae* (*p* < 0.001) (Figure 5B) of the top 20 families. Concordantly, genus-level shifts within the top 30 taxa comprised marked reductions in *14-2* (*p* < 0.01), *Romboutsia_B* and *Faecalibaculum* (*p* < 0.001) following IOP-H treatment (Figure 5D). These high-abundance, dose-responsive taxa may be the potential mediators of the protective effects conferred by IOP-H.

The linear discriminant analysis (LEfSe) further corroborated that IOP-H selectively depleted the taxa that were specifically enriched in the HFD group compared with the Control group (Figure 5E,F), most notably *Erysipelotrichaceae* and *Faecalibaculum*, which were positively related to dietary sugar and lipid uptake [33]. Moreover, LEfSe profiling at the species level revealed that IOP-H partially reversed HFD-induced dysbiosis by selectively depleting all five species that were enriched in HFD-fed mice relative to controls (Figure 5E,F). To identify the bacterial species that mediate this protective effect, we next applied a random forest model. Besides several uncultured taxa such as *UMGS1994 sp90055394*, *14-2 sp001940225* and *OLB9 sp001567255*, *Faecalibaculum rodentium* emerged as the highest-contributing discriminator, implicating it as a keystone species. Collectively, these data indicate that IOP-H mitigates HFD-associated dysbiosis largely through suppression of disease-associated bacteria—particularly *Faecalibaculum rodentium*. Collectively, these data establish that IOP-H exerts potent microbiota-modulating activity, an effect attributable—at least in part—to the selective suppression of HFD-associated pathogens, most notably *Faecalibaculum rodentium*, thereby resolving diet-induced dysbiosis.

### 3.6. The IOP-Altered Gut Microbes Are Associated with Obesity-Related Phenotypes

**Figure 6:**
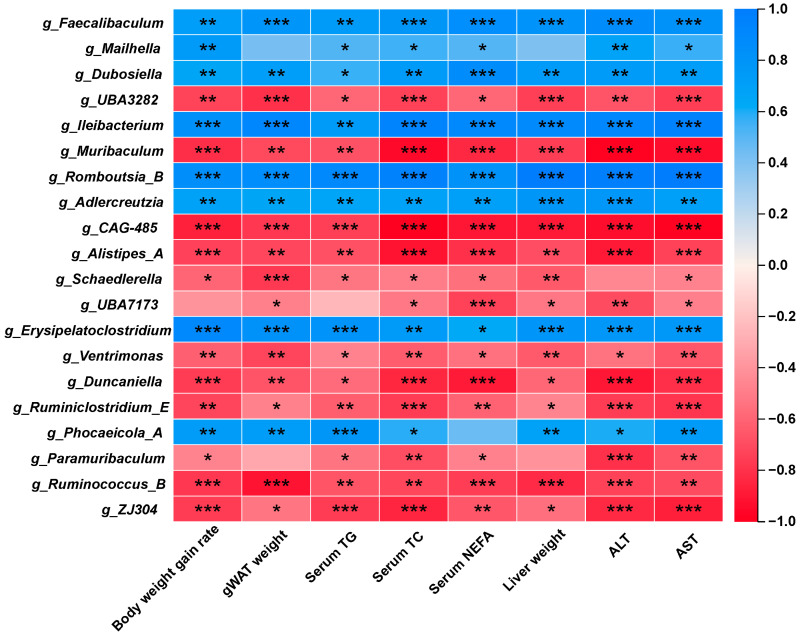
Correlation heatmap analysis between key gut microbiota genera and obesity-related indicators in HFD-induced obese mice. The color gradient represents the correlation coefficient, with blue indicating positive correlations and red indicating negative correlations. Asterisks denote statistically significant differences, * *p* < 0.05, ** *p* < 0.01, *** *p* < 0.001.

To further elucidate the intricate relationship between the altered gut microbiota and metabolic parameters, a genus-level correlation heat map was generated using Spearman’s rank correlation analysis (Figure 6). As illustrated in the results, a cluster of obesity-promoting bacteria—predominantly *g_Faecalibaculum* and *g_Romboutsia_B*—exhibited robust positive correlations with obesity-related indices (body weight gain rate and fat accumulation) and serum lipid markers (TG, TC and NEFA). This further confirms the ameliorative effect of those beneficial bacteria on obesity. Conversely, potential beneficial taxa, such as *g_Muribaculum*, *g_CAG-485* and *g_Alistipes A*, displayed distinct negative correlations with lipid profile markers and tissue weight index. This suggests that these genera may play a protective role in the progression of metabolic syndrome. Furthermore, this heatmap also indicated that the aforementioned beneficial and harmful bacteria are closely correlated with liver function indices (ALT and AST), suggesting that their compositional patterns contribute to the impairment of liver function. These correlation analyses provided robust evidence that alterations in the abundance of specific beneficial and harmful gut microbes are closely linked to the regulation of host metabolism and liver function.

## 4. Discussion

The rising global incidence of obesity and its related metabolic disorders, including metabolic dysfunction-associated steatotic liver disease (MASLD), has driven the search for effective natural therapeutic agents. In this study, we showed that intervention with *Inonotus obliquus* polysaccharides significantly alleviated high-fat diet-induced obesity and improved liver function in mice through modulation of the gut microbiota. These findings highlight its potential as a functional food ingredient for promoting metabolic health.

Chronic HFD consumption fundamentally disrupts the homeostatic balance between energy intake and expenditure, ultimately culminating in a cluster of metabolic syndromes, including excessive weight gain, ectopic lipid deposition, and systemic dyslipidemia [34]. In the present study, supplementation with 0.2% IOP effectively decoupled HFD intake from its characteristic pathological outcomes, as evidenced by the significant suppression of weight gain, as well as the marked reduction in visceral fat accumulation and serum lipid profiles (Figure 2 and Figure 3). These findings are highly consistent with a recent study by Li Yang et al., which reported that both low (800 mg/kg/d) and high (1000 mg/kg/d) doses of IOP intervention effectively alleviated adipose accumulation and hepatic injury in hyperlipidemic mice [17]. Notably, while the aforementioned study observed therapeutic efficacy at their tested concentrations, the 0.1% IOP group in our study (approximately 70 mg/kg/day based on average food intake) exhibited no significant anti-obesity effects. In contrast, the 0.2% dosage group (approximately 250 mg/kg/day) displayed potent bioactivity. This discrepancy in the low-dose group may be attributed to a concentration-dependent threshold and differences in the mode of administration. Unlike oral gavage, which triggers a rapid physiological response via a concentrated bolus, dietary incorporation (mixing with chow) ensures a sustained but lower peak luminal concentration. This suggests that a critical dietary intake level of IOP is essential to sufficiently modulate the intestinal environment or facilitate systemic lipid mobilization. Furthermore, the significant preventive effect achieved at the 0.2% dosage further indicates that the galactoglucan structure of IOP may serve as a key structural feature contributing to its lipid-lowering activity. Compared with previously reported studies on the anti-obesity effects of IOP, the excellent lipid-lowering efficacy of IOP observed here may be related to its smaller molecular weight (5.4 kDa), which might be more readily utilized by the gut microbiota. Furthermore, studies by Ling Su et al. (2022) and Yiming Guo et al. (2025) have corroborated that polysaccharides derived from *Inonotus obliquus* (IN and IOP) significantly improve lipid homeostasis in HFD/STZ-induced diabetic mice [19,35]. The consistent performance of IOP across diverse metabolic models—ranging from simple hyperlipidemia to complex diabetes—strongly reinforces its pharmacological robustness as a strategic intervention for metabolic syndrome.

The gut microbiota has a profound impact on host glucose homeostasis. High-fat diet (HFD)-induced obesity models are frequently accompanied by gut microbiota dysbiosis, characterized by reduced α-diversity and the enrichment of bacterial taxa such as Erysipelotrichaceae, which have been increasingly linked to metabolic dysfunction [36,37]. Consistent with previous reports, our findings revealed that HFD markedly suppressed the α-diversity of the gut microbiota and specifically enriched Erysipelotrichaceae. Notably, dietary supplementation with fungal polysaccharides can ameliorate HFD-induced metabolic disorders by restoring gut microbial diversity, thereby mitigating obesity and associated pathologies. In agreement with these observations, the present study demonstrated that IOP treatment elevated the Shannon diversity index and substantially reshaped the β-diversity landscape of the gut microbiota. Our data indicate that modulatory effect of IOP on intestinal microbial ecology may constitute an important mechanistic basis for its alleviation of HFD-induced host metabolic dysregulation.

As a high-molecular-weight biomacromolecule, the polysaccharide is poorly absorbed and utilized by the host. However, the gut microbiota encodes a vast repertoire of glycoside hydrolases that directly engage dietary polysaccharides, thereby altering the host’s health and disease status [38]. Our findings revealed that high-dose IOP administration was associated with an amelioration of HFD-induced dysbiosis, specifically by attenuating the expansion of *Faecalibaculum rodentium* (belonging to family Erysipelotrichaceae), a bacterial taxon previously identified to exhibit a significant positive correlation with high-fat/high-sugar diet [33]. Moreover, high-dose IOP-polysaccharide intervention was associated with reduced levels of potentially pathobiontic taxa and a relative enrichment of *Staphylococcus*. Notably, prior surveys have failed to disclose any specific association between *Staphylococcus* and HFD exposure. We therefore posit that *Staphylococcus* and *Faecalibaculum* might occupy overlapping ecological niches; the expansion of the former is a secondary consequence of IOP-mediated suppression of the latter rather than a direct, HFD-counteracting mechanism of IOP. Furthermore, in contrast to the previous literature, we observed that IOP treatment reduced the abundance of the potentially beneficial taxon *Akkermansia*. This observed reduction in *Akkermansia* should not be viewed as a simple contradiction but rather as a reflection of comprehensive ecological niche restructuring within the gut microbiome induced by IOP. The decrease in *Akkermansia* may be functionally compensated by the concurrent enrichment of other beneficial taxa, such as *Muribaculum* and *Alistipes_A*, which collectively contribute to maintaining gut ecological stability and metabolic homeostasis. This perspective of functional redundancy and niche complementarity provides an ecological framework to interpret the so-called “Akkermansia Paradox” in dietary interventions. Furthermore, these discrepant findings across studies might be attributable to strain-level heterogeneity within *Akkermansia*. The specific probiotic strains reported to increase in other studies may differ from those that decreased in the present study [39].

The oral bioavailability of polysaccharides has long been debated. A significant barrier has been the technical difficulty in tracing specific polysaccharides in biological matrices. However, recent work employing fluorescent and isotopic tracing directly demonstrated the intestinal absorption and systemic detection of specific mushroom β-1,3-glucans in rodents, primarily via clathrin-mediated endocytosis [40]. Based on structural similarity, IOP may also be absorbed via a similar pathway. The metabolic improvements upon IOP supplementation offer indirect evidence for its bioavailability. While direct measurement of unlabeled IOP remains challenging, future studies with labeled IOP are warranted to define its pharmacokinetics and translational potential as a functional food ingredient.

## 5. Conclusions

In summary, this study confirmed that IOP, a polysaccharide derived from *Inonotus obliquus*, reduced body weight, alleviated systemic lipid accumulation and ameliorated obesity-induced liver injury. IOP exerted a selective regulatory effect on the gut microbiota composition, significantly upregulating the abundance of *Streptococcaceae* at the family level while suppressing *Saprospiraceae*, *Peptostreptococcaceae*, *Erysipelotrichaceae* and *14-2*. At the genus and species levels, the abundances of *Romboutsia_B*, *Faecalibaculum* and *Faecalibaculum rodentium* were reduced. This study not only lays a solid foundation for developing IOP as a potential functional food ingredient, but also provides novel experimental evidence for mushroom polysaccharides to prevent and intervene in obesity through modulation of gut microbiota composition.

## Data Availability

The original contributions presented in this study are included in the article. Further inquiries can be directed to the corresponding authors.
